# Inhibition of mTORC1/C2 signaling improves anti-leukemia efficacy of JAK/STAT blockade in *CRLF2* rearranged and/or *JAK* driven Philadelphia chromosome–like acute B-cell lymphoblastic leukemia

**DOI:** 10.18632/oncotarget.24261

**Published:** 2018-01-17

**Authors:** Qi Zhang, Ce Shi, Lina Han, Nitin Jain, Kathryn G. Roberts, Helen Ma, Tianyu Cai, Antonio Cavazos, Yoko Tabe, Rodrigo O. Jacamo, Hong Mu, Yang Zhao, Jing Wang, Shuo-Chieh Wu, Fenglin Cao, Zhihong Zeng, Jin Zhou, Yingchang Mi, Elias J. Jabbour, Ross Levine, Sarah K. Tasian, Charles G. Mullighan, David M. Weinstock, David A. Fruman, Marina Konopleva

**Affiliations:** ^1^ Department of Leukemia, The University of Texas M.D. Anderson Cancer Center, Houston, TX, USA; ^2^ Department of Pathology, St. Jude Children's Research Hospital, Memphis, TN, USA; ^3^ Department of Next Generation Hematology Laboratory Medicine, Juntendo University School of Medicine, Tokyo, Japan; ^4^ Department of Bioinformatics & Comp Biology, The University of Texas M.D. Anderson Cancer Center, Houston, TX, USA; ^5^ Department of Medical Oncology/Hematologic Neoplasia, Dana-Farber Cancer Institute, Boston, MA, USA; ^6^ Department of Hematology, The First Hospital Affiliated Harbin Medical University, Harbin, China; ^7^ Department of Leukemia, Institute of Hematology & Blood Diseases Hospital, Chinese Academy of Medical Science & Peking Union Medical College, Tianjin, China; ^8^ Hematologic Malignancies, Memorial Sloan Kettering Cancer Center, New York, NY, USA; ^9^ Division of Oncology and Center for Childhood Cancer Research, The Children's Hospital of Philadelphia, Philadelphia, PA, USA; ^10^ Department of Molecular Biology & Biochemistry, University of California, Irvine, Irvine, CA, USA

**Keywords:** Ph-like ALL, JAK, mTOR

## Abstract

Patients with cytokine receptor-like factor 2 rearranged (*CRLF2*-re) subgroup Philadelphia chromosome–like B-cell acute lymphoblastic leukemia (Ph-like B-ALL) have a high relapse rate and poor clinical outcomes. *CRFL2*-re Ph-like B-ALL is characterized by heightened activation of multiple signaling pathways, including the JAK/STAT and PI3K/AKT/mTOR pathways. We hypothesized that the combined inhibition by JAK2 and mTOR inhibitors would induce an additive antileukemia effect in *CRLF2*-re Ph-like B-ALL. In this study, we tested the antileukemia efficacy of the type I JAK inhibitor ruxolitinib and type II JAK inhibitor NVP-BBT594 (hereafter abbreviated BBT594) [1] alone and combined with allosteric mTOR inhibitor rapamycin and a second generation ATP-competitive mTOR kinase inhibitor AZD2014. We found that BBT594/AZD2014 combination produced robust anti-leukemic effects in Ph-like cell lines *in vitro* and in patient-derived xenograft (PDX) cells cultured *ex vivo*. JAK2/mTOR inhibition arrested the cell cycle and reduced cell survival to a greater extent in Ph-like B-ALL cells with *CRLF2*-re and *JAK2* mutation. Synergistic cell killing was associated with the greater inhibition of JAK2 phosphorylation by BBT594 than by ruxolitinib and the greater inhibition of AKT and 4E-BP1 phosphorylation by AZD2014 than by rapamycin. *In vivo*, BBT594/AZD2014 co-treatment was most efficacious in reducing spleen size in three Ph-like PDX models, and markedly depleted bone marrow and spleen ALL cells in an *ATF7IP-JAK2* fusion PDX. In summary, combined inhibition of JAK/STAT and mTOR pathways by next-generation inhibitors had promising antileukemia efficacy in preclinical models of *CRFL2-*re Ph-like B-ALL.

## INTRODUCTION

Philadelphia chromosome–like B-cell lymphoblastic leukemia (Ph-like B-ALL) is a B-ALL subgroup of patients whose transcriptional profile resembles *BCR-ABL1*-positive B-ALL but does not contain an actual *BCR-ABL1* fusion [2]. This subgroup comprises 10-20% of children with high-risk pediatric B-ALL and nearly 30% of young adults with B-ALL [3]. Patients with Ph-like B-ALL experience high rate of relapse and have poor overall survival [4]. Novel therapeutic strategies are urgently needed to improve outcomes.

The vast majority of patients with Ph-like B-ALL harbor kinase-activating alterations, including chromosomal rearrangement, structural variations, and sequence mutations [3, 5], resulting in cytokine-independent cell proliferation and activation of multiple cell signaling pathways. Rearrangement of cytokine receptor-like factor 2 (*CRLF2*-re), resulting in *CRLF2* overexpression, is the most frequent alteration, occurring in about 50% of Ph-like B-ALL cases [3, 4]. The rearrangement links the full-length *CRLF2* coding region to transcription control elements driving elevated expression. Rearrangements include translocations to immunoglobulin heavy chain (*IGH*) locus enhancer elements or to the *P2RY8* promoter through intra-chromosomal deletion [6]. Approximately half of *CRLF2*-re cases harbor focal deletions or sequence mutations in JAK pathway–associated genes, including *IL7R*, *FLT3*, *SH2B3*, *JAK1*, and most frequently *JAK2* [7]. Activation of the JAK/STAT signaling pathway promotes cell survival, proliferation, and migration.

Constitutive activity of the JAK/STAT pathway may also facilitate upregulation of the downstream PI3K/AKT/mTOR pathway contributing towards ALL pathogenesis. The PI3K/AKT/mTOR signaling network is one of the most frequently activated pathways in human cancer and is subject to complex cross-talk and feedback. mTOR has two different kinase complexes: mTOR complex 1 (mTORC1, with RAPTOR) and mTORC2 (with RICTOR) [8]. The mTORC1 kinase complex binds RAPTOR to phosphorylate downstream proteins S6 kinase (S6K) and 4E-BP1, in turn regulating the cap-dependent mRNA translation of proteins that are critical for cell cycle progression from G1 to S phase [9]. The mTORC2 kinase complex binds RICTOR to phosphorylate AKT on Ser473, regulating cell survival [8].

Type I JAK2 inhibitors such as ruxolitinib bind within the ATP-binding pocket of the active conformation of JAK2, to compete with ATP, thereby inhibiting the phosphorylation of STAT5 and p-STAT5 signaling within tumor cells. However, such effects have limited effect on survival of ALL cell lines and in patient-derived xenograft (PDX) models [10, 11]. Recent work has already proposed favorable combination of JAK2 inhibition and PI3K/mTOR inhibition [11]. However, first generation mTOR inhibitors such as rapamycin only partially inhibit mTORC1, thus only transiently inhibiting 4E-BP1 phosphorylation [12] and do not affect mTORC2/AKT. On the contrary, inhibition of S6K can activate p-AKT through a feedback mechanism which promotes cell survival [13].

This led to our hypothesis that combined inhibition of both JAK/STAT and mTOR pathways with next generation kinase inhibitors may be beneficial in patients with *CRLF2*-re or *JAK2* driven Ph-like B-ALL. To test this hypothesis, we utilized BBT594, a dihydroindole pharmacophore type II inhibitor originally developed as an inhibitor of the T315I BCR-ABL mutant, which also inhibited other isoforms of BCR-ABL and receptor tyrosine kinase such as JAK2 [1] and RET [14]. As a type II inhibitor, upon binding BBT594 extends to a hydrophobic site adjacent to the ATP-binding site of JAK2 thereby stabilizing the kinase in an inactive conformation, preventing phosphorylation of the activation loop [15]. Transphosphorylation of JAK2 by JAK1 or TYK2 does not confer resistance to BBT594 [16], and CHZ868, a benzimidazole analogue JAK2 type II inhibitor, was recently shown to have superior activity in blocking JAK2 signaling in Ph-like cell line model BaF/3 expressing Ph-like related genes and murine transgenic Ph-like ALL model co-expressing *CRLF2* and mutant *JAK2* R683G [15]. We further utilized second generation mTOR inhibitor AZD2014 that targets both mTORC1 and mTORC2. In this study, we examined combined efficacy of these agents in human and murine engineered cell lines harboring rearranged *CRLF2* and/or *JAK2* mutation and in patient-derived Ph-like xenografts *in vitro* and *in vivo*.

## RESULTS

### Combined inhibition of JAK2 and mTOR induced synergistic inhibition of cell growth in cell lines with *CRLF2* rearrangement and *JAK2* mutation

**Figure 1:**
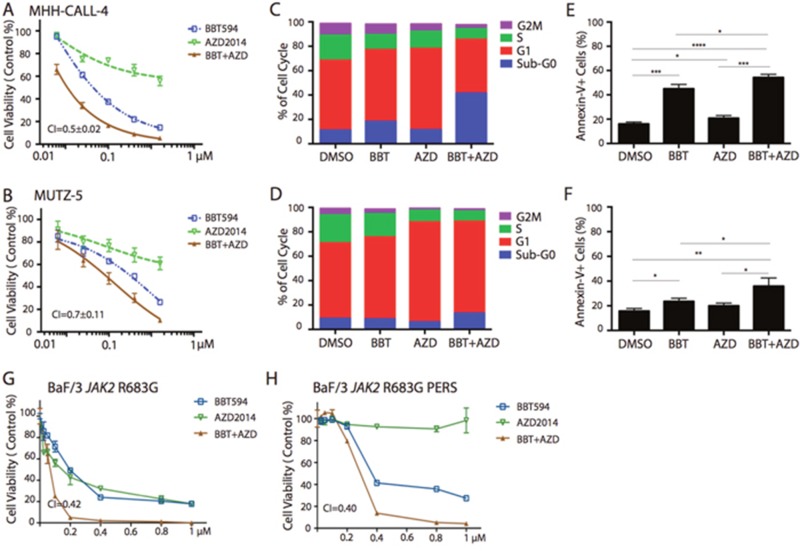
*In vitro* antileukemia efficacy of dual JAK2 and mTOR inhibition in Ph-like B-ALL cell lines MHH-CALL4 and MUTZ-5 cells were treated with 0.25-0.8μM BBT594 (BBT), AZD2014 (AZD), or combinations for 72 h, then the numbers of viable cells were determined by CTG assay. The cell inhibition curves were plotted with the live cell number normalized to those of DMSO-treated controls: **(A)** MHH-CALL4 cells, **(B)** MUTZ-5 cells. Treated cells were fixed in 90% methanol and then stained with propidium iodine to determine the effects of the treatments on the cell cycle by flow cytometry: **(C)** MHH-CALL4 cells, **(D)** MUTZ-5 cells. The cells were stained with annexin V/DAPI to quantify cell apoptosis by flow cytometry: **(E)** MHH-CALL4 cells, **(F)** MUTZ-5 cells. ^*^ p<0.05, ^**^ p<0.005, ^***^ p<0.0005 as determined by unpaired Student t-test. **(G, H)** BaF/3 cells expressing the Ph-like B-ALL-associated JAK2 R683G parental (G) and ruxolitinib-“persistent” cells (H) were treated with 0.25-1.0μM BBT594, AZD2014 or the combination for 72 h, and cell viability was analyzed by CTG assay. The viability of cells treated was selected inhibitors was to those of DMSO-treated controls, and expressed as % viable cells.

We first evaluated the effects of JAK2 and mTOR inhibitors in two *JAK2*-driven human Ph-like B-ALL cell lines: MHH-CALL-4, which harbors *IGH-CRLF2* translocation and the *JAK2* I682F mutation, and MUTZ-5 with *IGH-CRLF2* translocation and the *JAK2* R683G mutation. Cell line REH, which harbors *ETV6-RUNX1* translocation and is wild-type for *CRLF2* and *JAK2*, served as a negative control. In the proliferation assay, ruxolitinib used at a concentration range between 0.025μM and 0.4μM had minimal or no effect on all tested cell lines (Supplementary Figure 1A-1B). In contrast, the same concentration range of type II JAK2 inhibitor BBT594 potently inhibited growth of *JAK2*-driven cell lines with an IC_50_ of 0.1 ± 0.005 μM in MHH-CALL-4 and 0.1± 0.008 μM in MUTZ-5 (Figure 1A-1B). In non–*JAK2*-driven REH cells, only high concentrations exceeding 1μM of BB T594 showed minimal growth-inhibitory effects, possible due to off-target activity (Supplementary Figure 1C). Rapamycin alone or when combined with ruxolitinib had very modest growth-inhibitory effects in MHH-CALL-4 and MUTZ-5 cells (Supplementary Figure 1A-1B). In contrast, AZD2014 caused more pronounced growth inhibition in all three cell lines, and the combination of ruxolitinib and AZD2014 was synergistic in *JAK2*-mutated cells (CI_s_ 0.7±0.3 and 0.3±0.1, respectively) but not in REH cells (Figure 1A-1B, Supplementary Figure 1A-1C). BBT594 showed additive/moderate synergy when combined with rapamycin (CI_s_ 0.4± 0.03 and 0.9± 0.02) (Supplementary Figure 1A-1B), and strong synergy upon combined use with AZD2014 (CI_s_ 0.5±0.02 and 0.7± 0.11) (Figure 1A-1B) (summarized in Table 1).

**Table 1:** Combination Index (CI) of the compound combinations in MHH-CALL-4 and MUTZ-5 cell lines

CI	MHH-CALL-4		MUTZ-5	
	Rapamycin	AZD2014	Rapamycin	AZD2014
Ruxolitinib	148.6±166.3	0.7±0.3	0.6±0.6	0.3±0.1
BBT594	0.4±0.03	0.5±0.02	0.9±0.02	0.7±0.11

Compared to the control REH cell line, in which only mTOR inhibitors affected cell cycle distribution, the combinations of a JAK2 inhibitor and an mTOR inhibitor arrested the MHH-CALL-4 and MUTZ-5 cells in the G1/sub-G0 phase with concomitant reductions in the percentages of cells in G2M/S phase (Figure 1C-1D, Supplementary Figure 1D-1F). While ruxolitinib alone or in combination with mTOR inhibitors failed to or induced only minimal cell death, BBT594 used as a single agent and more so in combination with AZD2014 induced modest apoptosis in MHH-CALL-4 and MUTZ-5 cells (Figure 1E-1F, Supplementary Figure 1G-1I).

### Combined inhibition of JAK2 and mTOR induced synergistic inhibition in BaF/3 cell lines with Ph-like B-ALL–associated *JAK* mutations

We next compared efficacy of ruxolitinib or BBT594 in combination with AZD2014 in parental or ruxolitinib-“persistent” BaF/3 cells expressing the Ph-like B-ALL–associated *JAK1* V658F mutation, the *JAK2* R683G mutation, or co-expressing wild-type *CRLF2* and the *JAK2* R683S mutation, described previously [15, 17]. In these cells generated by chronic exposure to ruxolitinib, JAK2 is stabilized and reactivated secondary to heterodimeric association of JAK2 with JAK1/TYK2 and JAK2 transactivation [16]. Three parental BaF/3 cells engineered to express *JAK1* V658F and *JAK2* R683G mutations with or without *CRLF2* were sensitive to inhibitors, and combined inhibition of JAK and mTOR synergistically inhibited cell growth in all except *JAK2* R683G cells (Figure 1G and Supplementary Figure 2A-2C). As reported, ruxolitinib-“persistent” cells were resistant to up to 8μM ruxolitinib, while the median inhibitory concentration (IC_50_) was 0.2 to 0.8 μM in the parental cells. In turn, BaF/3 *JAK1* V658F and BaF/3 *CRLF2*/*JAK2* R683S (but not *JAK2* R683G) “persistent” cells remained sensitive to mTOR C1/2 inhibitor AZD2014, which in combination with ruxolitinib induced higher apoptosis in these cells (*p*=0.01 and *p*=0.007) (Figure 1H and Supplementary Figure 2A-2C).

Furthermore, even though variably, ruxolitinib-“persistent” cells responded to BBT594, and there was synergistic cell inhibition when exposed to BBT594/AZD2014 combination (Figure 1H and Supplementary Figure 2A-2C). These data suggest that combinations of JAK2 and mTOR inhibitors induce synergistic antileukemia activity in Ph-like ALL cells, and that blockade of mTOR signaling can partially reverse resistance to ruxolitinib, further enhancing sensitivity to BBT594.

### JAK2 and mTOR inhibitors reduce TSLP-stimulated signaling in Ph-like ALL cells

**Figure 2:**
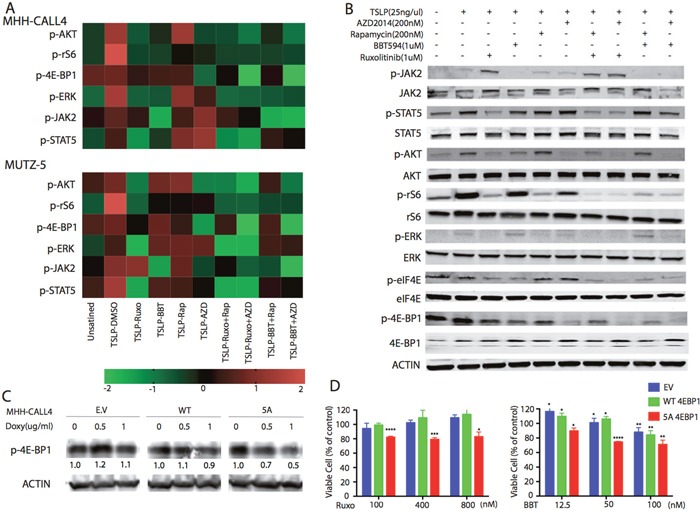
Inhibitory effects of dual JAK2 and mTOR inhibition on the PI3K/mTOR pathway **(A)** Phospho-flow cytometry analysis demonstrated increased phosphorylation of JAK-2(Tyr1008), STAT5(Ty694)), ERK(T202/Y204) and AKT/pS6 [AKT(Ser473)],4E-BP1(T37/46), and rS6(S240/244) after stimulation with TSLP(25ng/mL) for 30 min. MHH-CALL4 and MUTZ-5 cells were treated with indicated compounds for 1 h, then the phosphorylation signals of PI3K/mTOR pathway proteins were determined by phospho-flow cytometry. Heat map data depict the changes in phosphoprotein levels by median fluorescence intensity. Color scale represents normalization (Z-score) of each condition for each signal. **(B)** MHH-CALL4 cells were stimulated with TSLP for 30min followed by treatment with ruxolitinib (Ruxo), BBT594 (BBT), rapamycin (Rap), or AZD2014 (AZD) or one of their combinations at the indicated doses for 1 h, after which the expression of PI3K/mTOR pathway proteins were probed by Western blot. **(C)** MHH-CALL4 cells were infected with empty vector (EV), with a wild-type (WT) or with mutant (5A) 4E-BP1 constructs, then treated with indicated concentrations of doxycycline (Doxy) for 72 h to induce p-4E-BP1 expression. Levels of p-4E-BP1 were determined by Western blot. **(D)** MHH-CALL4 cells transfected with 4E-BP1 constructs were treated with doxycycline (1μg/mL) for 72 h to induce 4E-BP1 and then with ruxolitinib (Ruxo) or BBT594 (BBT) for 72 h. Cell viability was analyzed by CTG assay. ^*^ p<0.05, ^**^ p<0.005, ^***^ p<0.0005, ^****^ p<0.0001 as determined by unpaired Student t-test.

We next examined the effects of these inhibitors on components of JAK/STAT and PI3K/mTOR pathways using phospho-flow cytometry. In these experiments, we utilized a recombinant ligand of CRLF2, thymic stromal lymphopoietin (TSLP), to simulate conditions of the BM microenvironment [18]. TSLP robustly stimulated the phosphorylated (p)-rS6, p-ERK, and p-JAK2 signals and moderately induced the p-AKT, p-STAT5, and p-4E-BP1 signals in MHH-CALL-4 or MUTZ-5 cells (Figure 2A). Ruxolitinib inhibited TSLP-induced but not constitutive (baseline) p-JAK2 and reduced p-STAT5 below baseline levels. In turn, BBT594 fully inhibited both, constitutive and TSLP-induced p-JAK2 and inhibited p-STAT5 to the baseline level. Rapamycin inhibited p-rS6 and p-4E-BP1, but increased levels of p-AKT, while AZD2014 fully inhibited all three phospho-proteins. Neither rapamycin nor AZD2014 fully inhibited p-ERK, while both JAK2 inhibitors blocked TSLP-induced p-ERK activation. The combinations of ruxolitinib+rapamycin and ruxolitinib+AZD2014 fully inhibited all the measured signals except for p-JAK2. In comparison, the combinations of BBT594+ rapamycin and BBT594+ AZD2014 fully inhibited p-JAK2 and other signaling, albeit less potently reducing p-STAT5 level. In turn, rapamycin alone or in combination failed to fully inhibit p-4E-BP1, while this was successfully achieved by AZD2014 in combinations with either JAK2 inhibitor.

The flow cytometry results were validated by immunoblotting in MHH-CALL-4 cells (Figure 2B). TSLP stimulation increased phosphorylation of all tested phospho-proteins. BBT594 completely inhibited p-JAK2, and AZD2014 inhibited p-AKT and p-4E-BP1 to a greater extent than rapamycin.

Since more efficient blockade of mTORC1, in particular 4E-BP1, has been implicated in the higher activity of next generation TORC inhibitors, we engineered MHH-CALL-4 cells to express doxycycline-inducible mutant 4E-BP1 5A [19]. Phosphorylation of 4E-BP1 is known to disrupt the complex between 4E-BP1 and the translation factor eIF4E, releasing free active eIF4E [20]. Phosphorylation-defective mutant 4E-BP1 5A was reported to tightly associate with eIF4E [19], inhibiting its translation activation and signaling functions downstream of 4E-BP1, mimicking effects of AZD2014 (as shown by decreased p-4E-BP1 level after doxycycline induction in Figure 2C). While there was no toxicity of 0.1ug/ml of doxycycline when 5A 4EBP1 was induced up to 72h (not shown), growth of cells induced to express a mutant non-phosphorylatable 4E-BP1 5A was further inhibited by BBT594 and by ruxolitinib compared to cells transfected with empty vector or wild-type plasmid constructs (Figure 2D).

**Figure 3:**
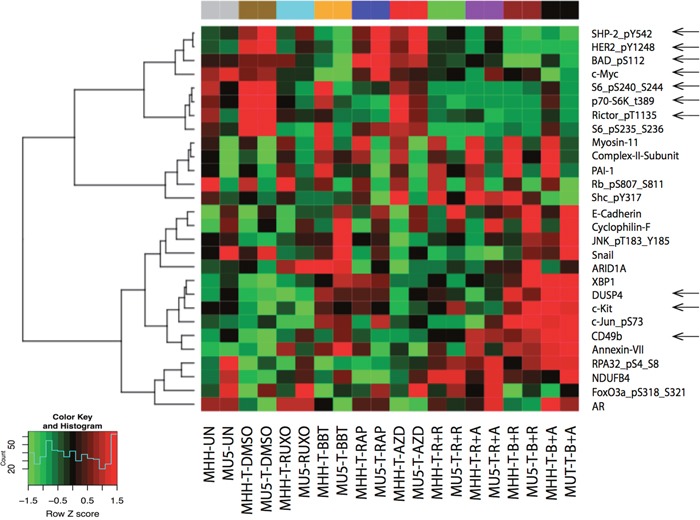
Inhibitory effects of dual JAK2 and mTOR inhibition by Reverse Phase Protein Array (RPPA) analysis MHH-CALL4 and MUTZ-5 cells were stimulated with TSLP for 30 min, treated with indicated compounds for 1 h, after which the cell lysates were prepared for RPPA analysis. The heatmap showed the differentially expressed proteins in at least on treatment group with adjust ANOVA *p* (FDR) < 0.1. Pearson distance metric and Ward's minimum variance was used to cluster the samples and proteins.

To explore additional mechanisms underlying the synergy, we performed the reverse phase protein array (RPPA) in MHH-CALL-4 and MUTZ-5 cells (Figure 3). In addition to anticipated changes in p70-S6K-Thr389, p-S6, and phospho-rictor (on T1135, an S6K1 site), ruxolitinib and BBT594 combined with AZD2014 decreased c-myc expression and phospho-Bad (Bad_pS112), both of which are targets of mTOR and JAK/STAT networks [21]. TSLP stimulated samples showed high level of tyrosine-protein phosphatase non-receptor type 11 (PTPN11, SHP2), human epidermal growth factor receptor 2 (HER2), which were in turn inhibited by JAK inhibitor and further reduced by combinations. On the other hand, TSLP stimulation decreased the level of CD49, stem cell factor receptor kinase (c-KIT), and dual specificity phosphatase 4 (DUSP4), which were strongly elevated in samples treated with combination of inhibitors. These findings indicate cross-talk with other pathways which could conceivably contribute to the synergy effect, to be explored in future studies.

### Combination of JAK type II inhibitor BBT594 with an mTOR inhibitor induced synergistic cell apoptosis in Ph-like B-ALL PDX samples *ex vivo*

To extend the cell killing and cell signaling *in vitro* findings to samples from patients with Ph-like B-ALL, we developed multiple PDX models of Ph-like B-ALL (Table 2). Notably, a subset of *CRLF2*-re/*JAK2* wild-type PDX cells (four of the seven tested) failed to grow *ex vivo* and were excluded from subsequent analysis. All PDX samples harboring *CRLF2*-re and *JAK2* mutation (four with *P2RY8-CRLF2* and three with *IGH-CRLF2*) and one sample with wild-type *CRLF2* and a *JAK2* fusion maintained viability *ex vivo* and were tested with the JAK2/mTOR inhibitors.

**Table 2:** Genomic information of Ph-like ALL PDX samples

PDX	Ph-like ALL Genomic Lesions	
	*CRLF2*	*JAK2*
1	*IGH-CRLF2*	WT
2	*P2RY8-CRLF2*	WT
3	*P2RY8-CRLF2*	WT
4	*IGH-CRLF2*	WT
5	*IGH-CRLF2*	WT
6	*IGH-CRLF2*	WT
7	*IGH-CRLF2*	WT
8	*P2RY8-CRLF2*	T875N
9	*IGH-CRLF2*	R683G
10	*IGH-CRLF2*	R683S
11	*P2RY8-CRLF2*	R683G
12	*IGH-CRLF2*	R683S
13	*P2RY8-CRLF2*	R683S
14	*P2RY8-CRLF2*	R683G
15	N/A	*PAX5-JAK2*
16	N/A	*ATF7IP-JAK2*

PDX 13, 14 were confirmed strong Ph-like signature by microarray and prediction analysis of microarray, which reported previously as SJBALL021755 and SJBALL191 [3].

**Figure 4:**
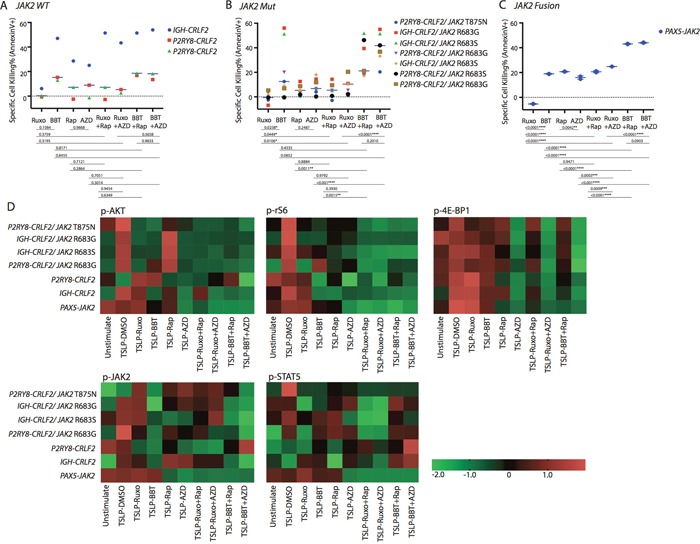
Antileukemia efficacy of dual JAK2 and mTOR inhibition *ex vivo* in Ph-like B-ALL patient-derived xenograft (PDX) models Cells collected from Ph-like B-ALL PDX models were treated with DMSO, ruxolitinib (Ruxo; 1 μM), BBT594 (BBT; 1 μM), rapamycin (Rap; 0.2 μM), AZD2014 (AZD; 0.2 μM), or one of their combinations for 48 h. The cells were then analyzed for **(A-C)** apoptosis by annexin V/DAPI or (D) the mTOR and JAK2/STAT phosphoproteins gated on CD19+ cells. **(D)** Heat map data depict the changes in phosphoprotein levels by fluorescence median intensity. Color scale represents normalization (Z-score) of each condition for each signal.

In three *CRLF2-*re/*JAK2* wild-type samples, ruxolitinib had no effect on cell death, while BBT594 alone or combined with rapamycin/AZD2014 induced modest cell apoptosis in 2 *P2RY8-CRLF2* samples and significant cell death in *IGH-CRLF2* sample *ex vivo* without notable synergistic effects with mTOR inhibitors (Figure 4A).

In the *CRLF2*-re/*JAK2-*mutated samples, BBT594 achieved significantly greater cell apoptosis than any other compound. The combinations of BBT594 with either mTOR inhibitor caused additional cell killing in the majority of samples tested (Figure 4B). In turn, ruxolitinib alone had no effect, while ruxolitinib+ AZD2014 showed additive induction of cell death (*p*=0.010). Similar results were seen in a single sample with a *JAK2* fusion (Figure 4C).

### Combined inhibition of JAK2 and mTOR achieved greater cell signaling inhibition in Ph-like B-ALL PDX models *ex vivo*

Phospho-signaling studies in samples with *CRLF2-*re with or without *JAK2* mutation showed findings consistent with cell lines studies, i.e. greater inhibition of AKT/mTOR outputs upon JAK2/mTOR inhibitors combinations, whereby only AZD2014-based combinations were capable of inhibiting p-4E-BP1 (Figure 4D). BBT594 fully inhibited constitutive or TSLP-induced p-JAK2 in all *CRLF2*-re samples, while ruxolitinib incompletely reduced p-JAK2 in 3 PDX and failed to do so in 3 additional PDX; co-treatment of BBT594 and AZD2014 had most profound inhibition of p-JAK2 in all samples tested. The combinations with ruxolitinib produced greater inhibition of p-STAT5, which was consistent with cell line data. In PDX with *PAX5-JAK2* fusion, ruxolitinib or BBT594 alone failed to inhibit either AKT/mTOR outputs or p-JAK2 but this was accomplished in combinations with either rapamycin or AZD2014, which on their own suppressed p-JAK2.

### Combination treatment in PDX models *in vivo*

**Figure 5:**
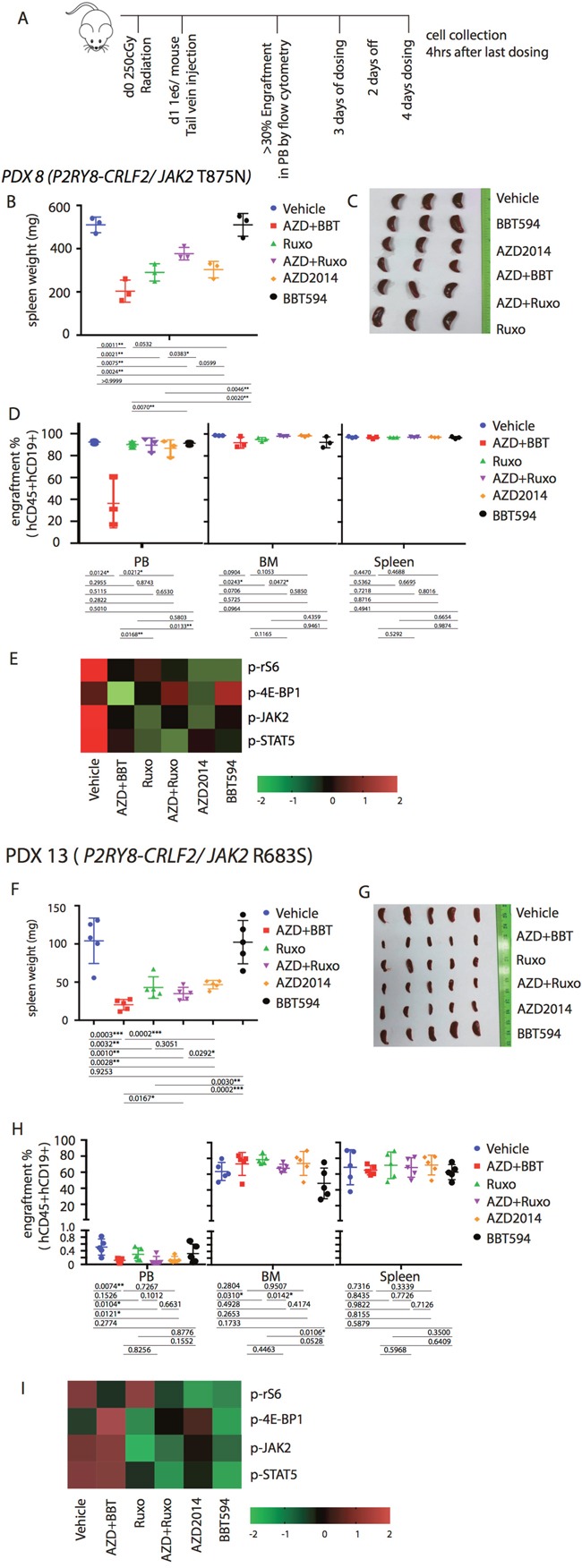
Anti-leukemia efficacy of dual JAK2 and mTOR inhibition *in vivo* in the *P2RY8-CRLF2*/*JAK2* mutation PDX **(A)** Treatment schema for *in vivo* experiment with PDX 8 (*P2RY8-CRLF2/JAK2* T875N) and PDX 13 (*R2RY8-CRLF2/JAK2* R683S). Mice were treated with vehicle, ruxolitinib (Ruxo, 2gm/mouse/day) BBT594 (BBT; 100mg/kg/d), AZD2014 (AZD; 30 mg/kg/d), or the combinations when higher than 30% leukemia cells engraftment was detected in peripheral blood (PB) for a total of 7 days (3 days on, 2 days off, 4 days on). 4 h after the last dosing was completed, mice were sacrificed for tumor burden assessment. Human leukemia cell percentages in the peripheral blood, bone marrow (BM), and spleen were measured by quantitative flow cytometry. Tumor burden was represented by spleen weight and morphology **(B, F, C** and **G)** and fraction of engraftment **(D, H)**. Heat map data depict the changes in phosphoprotein levels by fluorescence median intensity **(E, I)**.

**Figure 6:**
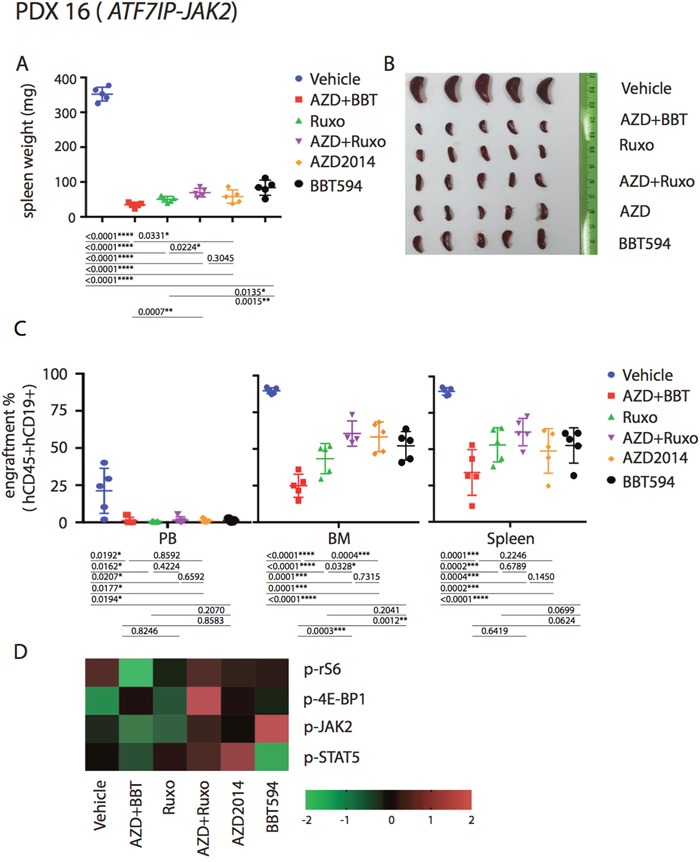
Anti-leukemia efficacy of dual JAK2 and mTOR inhibition *in vivo* in the *ATF7IP-JAK2* fusion PDX Mice were injected with PDX 16 (*ATF7IP-JAK2*) and treated as indicated in Figure 5A. Tumor burden was represented by spleen weight and morphology **(A, B)**, engraftment **(C)** and signaling **(D)**.

Based on the *in vitro* and *ex vivo* data, we hypothesized that the combinations of a type II JAK2 inhibitor and an mTOR inhibitor would achieve better anti-leukemia control in the *in vivo* Ph-like B-ALL PDX models. For these studies, we selected three Ph-like B-ALL PDX models, two *CRLF2*-re (*P2RY8-CRLF2/JAK2* T875N*, P2RY8-CRLF2*/*JAK2* R683S), and one with *ATF7IP-JAK2* fusion. Mice engrafted with ALL PDX were dosed for a total of 7 days with ruxolitinib, BBT594, AZD2014 or combinations as indicated in Figure 5A, after which engraftment in spleen, PB or BM was compared and spleen weight recorded. All mice tolerated therapy without significant weight loss (not shown). In both *CRLF2*-re PDX, spleen weights were significantly reduced by ruxolitinib alone or when combined with mTOR inhibitors, and most pronounced in the BBT594/AZD2014 combination group, indicating significant reduction of total tumor burden (Figure 5B, 5F). In contrast, the relative fraction (%) of ALL cells was minimally affected by all treatments with exception of decrease in PB ALL cells in the AZD2014/BBT594 combination group (Figure 5D, 5H). BBT594 at selected doses/duration did not significantly affect engraftment and spleen weights in *CRLF2*-re PDX. AZD2014 reduced spleen weights but did not affect engraftment. In stark contrast, in the *ATF7IP-JAK2* fusion model all inhibitors drastically reduced spleen weights and also significantly decreased fractions of leukemic cells in PB, BM and spleen, with BBT594/AZD2014 exhibiting most profound effect (Figure 6A-6D). In turn, ruxolitinib/AZD2014 combination failed to show better efficacy than ruxolitinib alone in any of the three models tested.

We next analyzed the signaling perturbations by JAK2 and mTOR inhibitors in the leukemia cells (gated on hCD19 by flow cytometry) collected from the murine spleens (Figure 5E, 5I, Figure 6D). *Ex vivo* signaling results exhibited differences compared to *in vitro* findings, possibly due to the dynamic signaling changes *in vivo* that cannot be fully reflected by single time point measurements; these inconsistences were noted in prior studies [11, 18]. For example, in *P2RY8-CRLF2*/*JAK2* R683S model BBT594/AZD2014, and in *ATF7IP-JAK2* model ruxolitinib/AZD2014 combination increased rather than decreased p-4E-BP1. In *P2RY8-CRLF2/JAK2* T875N model, both JAK2 inhibitors and additionally AZD2014 reduced p-JAK2, consistent with *in vitro* results. Ruxolitinib had stronger impact on p-STAT5 compared with BBT594. In *ATF7IP-JAK2* fusion model, BBT594 failed to inhibit p-JAK2, while AZD2014 alone and more so when combined with BBT594 or ruxolitinib, fully blocked p-JAK2. AZD2014 inhibited p-4E-BP1 and p-S6 (Figure 5E, 5I). In turn, greater combination effects of BBT594/AZD2014 were observed on p-S6 and p-STAT5 phospho-signals in the *ATF7IP-JAK2* fusion model (Figure 6D).

## DISCUSSION

Several prior studies have highlighted abnormal activation of PI3K/AKT/mTOR and JAK/STAT pathways in childhood Ph-like ALL models [18] and have demonstrated improved anti-leukemia efficacy upon combining type I JAK1/2 inhibitor ruxolitinib and PI3K/mTOR inhibitors [11, 18, 22]. While allosteric mTOR inhibitors such as rapamycin or its analogues have limited efficacy in various cancers in part due to activation of AKT signaling [23–26], second generation mTOR inhibitors can avoid the compensatory activation and elicit deeper inhibition of both mTOR complexes and downstream signaling [13, 27–29]. Further, recent studies in MPN and Ph-like ALL models have demonstrated superior activity of type II JAK2 inhibitors that fully inhibit p-JAK2 activity, and showed collateral activation of p-JAK2 as a mechanism of acquired resistance to ruxolitinib [15, 30].

In this study, we evaluated the combinatorial activity of the second generation mTOR inhibitor AZD2014 and of the type II JAK2 inhibitor BBT594 compared with allosteric mTOR inhibitor rapamycin and type I JAK2 inhibitor ruxolitinib in Ph-like ALL harboring *CRLF2* rearrangement. Studies in *CRLF2-*re*/JAK2*-mutated ALL cell lines, engineered BaF/3 cells or primary ALL PDX cultured *ex vivo* showed very little activity of ruxolitinib at sub-micro molar concentrations used. While rapamycin-based combinations elicited no further increase in anti-leukemia efficacy, combinations of ruxolitinib and the mTORC1/2 inhibitor AZD2014 were synergistic in human ALL cell lines and in BaF/3 cells including those which acquired resistance to ruxolitinib (ruxolitinib-“persistent” cells). It is notable that the reduction of viability based on CTG assay appears more prominent than effects on cell cycle or apoptosis induction. In this study, CTG assay was used across multiple inhibitor concentrations, cell cycle and induction of cell death was only examined at selected doses. It is possible, however, that the inhibitors directly affect cellular metabolism, especially in light of mTOR inhibition known to directly affect glucose uptake and glycolysis [31].

As anticipated from prior studies, AZD2014 unlike rapamycin fully suppressed activity of both mTOR complexes, including reduction of mTORC1 outputs with diminished p-4E-BP1, and of mTORC2 with a decrease in p-AKT. Cells expressing the 4E-BP1 5A mutant that suppresses eIF4E-dependent translation became more responsive to JAK2 inhibition. Albeit the reduction in viability was statistically significant, the extent of reduction was relatively minor. These data support the contributory, but not the dominant, role of mTOR/4E-BP1 in the observed synergy between JAK2 inhibitors and mTORC1/2 inhibitor AZD2014. In primary ALL PDX cultured *ex vivo* ruxolitinib/AZD2014 showed mainly additive effects that were limited to samples harboring mutant *JAK2* (Figure 4). It should be considered that the co-culture with stromal cells used in this study could have reversed cytotoxicity in the *JAK* WT cells and modified the outcomes of targeted therapies. Further, *in vivo* data in 2 PDX *CRLF2*-re/*JAK2*-mutated samples failed to show synergy of this combination, at least upon 7-day treatment regimen used (Figure 5), consistent with recently published data [11]. Another interesting finding is the anti-leukemia effect of ruxolitinib which was observed *in vivo* but not *in vitro* in PDX models, likely due to better proliferation of ALL PDX cells under nurturing conditions of murine microenvironment. In this study, we utilized 7-day treatment regimen and analyzed impact on tumor burden as end-point analysis, which prohibited survival assessments and remains a limitation of this pilot study. Curiously, we observed impressive reduction of spleen weights despite similar levels of engraftment. This could be related to the higher TSLP production in spleen reported by others [32], which triggers signaling in the *CRLF2*-re ALL cells, making them more vulnerable to the target inhibition. Overall, these findings indicate potential utility of second generation mTOR inhibitor/ruxolitinib combinations in *CRLF2*-re/*JAK2*-mutated Ph-like ALL, but additional *in vivo* studies using prolonged regimens of drug administration are needed to support this premise.

Our results strongly indicate superior activity of the type II JAK2 inhibitor BBT594, alone and more so when combined with AZD2014. This was demonstrated in both human and murine cell lines, including ruxolitinib-persistent BaF/3 cells which resistant up to 8μM ruxolitinib, and in *JAK2*-mutated ALL PDX samples cultured *ex vivo*. Analysis of intracellular signaling demonstrated superior ability of BBT594 to inhibit p-JAK2, while exhibiting less profound blockade of p-STAT5. Similar results were seen upon analysis of spleen cells from mice co-treated with BBT594 and AZD2014 (Figure 5). These findings indicate that other downstream JAK2 targets might be important for the observed anti-leukemia efficacy of BBT594. Deeper inhibition of JAK2 by BBT594 was also reported previously in Ph-like murine model [15] and in MPN murine models [1, 16]. Less likely, off-target potency against other kinases could contribute towards superior activity of BBT594, however none of the identified targeted kinases [15] have a known role in ALL survival. While these need further detailed studies, our preliminary RPPA studies demonstrated inhibition of c-myc and decreased levels of phosphorylated, i.e. inactivated, apoptotic protein Bad, both of which play an important role in cellular proliferation and survival. Induction of HER2 expression after TSLP stimulation in MHH-CALL4 and MUTZ-5 cells might indicate possible trastuzumab sensitivity which needs further study. Altogether, these results indicate superior activity of type II JAK2 and second generation mTOR inhibitors in *CRLF2*-re/*JAK2*-mutated Ph-like ALL, secondary to more profound inhibition of collateral JAK2 and 4E-BP1 signaling.

In our study, a single model with *JAK2* fusion Ph-like ALL was evaluated. Consistent with previous studies [10], this PDX was more sensitive to ruxolitinib *in vivo* but not *in vitro* (Figure 4C and Figure 6A-6D). However, combination treatment with BBT594 and AZD2014 produced highest activity both in *ex vivo* and *in vivo* study, with 65%, 56% and 20% reduction in the fraction of ALL cells in BM, spleen, and PB, respectively (Figure 6C). Importantly, only combination of JAK2 inhibitors with mTOR inhibitors elicited full inhibition of p-JAK2, p-STAT5 and p-4E-BP1, consistent with previous findings that mTOR inhibitors could reduce activation of JAK/STAT pathway, ie. p-STAT5 [18, 22, 33]. Curiously, mTOR inhibitor reduced p-JAK2 in this model, invoking a possibility of mTOR pathway mediated phosphorylation of PAX-5 portion of the mutant fusion proteins, in turn controlling the dimerization and activation of PAX5-JAK2. Importantly, these fusions are observed more commonly in the adolescent and young adult (AYA) population and in adult Ph-like ALL and represent an important object for future studies [3, 34].

In summary, our findings indicate that the combined inhibition of the JAK/STAT and mTOR pathways by novel more potent inhibitors can effectively inhibit cell signaling and reduce leukemia burden in *CRFL2*-re Ph-like B-ALL cell lines and PDX models. Therefore, concurrent targeting of both pathways is a promising new therapeutic strategy for patients with *CRFL2-*re Ph-like B-ALL. While type II JAK2 inhibitors are not currently in clinical development, other strategies aimed at complete inhibition of JAK2 (such as HSP90 inhibitors) may produce greater anti-leukemia efficacy than ruxolitinib in *CRLF2*-re Ph-like ALL [35, 36].

## MATERIALS AND METHODS

Information concerning cell origin, authentication, culture, reagents, proliferation and cytotoxicity assays, phospho-flow cytometry, Western blotting and reverse phase protein array (RPPA) are detailed in the Supplementary Materials.

### PDX model establishment and *ex vivo* experiments

All samples were obtained with patient or parent/guardian provided informed consent under protocols approved by the Institutional Review Board at University of California-San Francisco Medical Center, Dana-Farber Cancer Institute, St. Jude Children's Research Hospital and M.D. Anderson Cancer Center. PDX 13-16 were already published previously [3]. PDX 13 and 14 with *P2RY8-CRLF2* expressed strong Ph-like signature by gene expression microarray, and were previously reported as SJBALL021755 and SJBALL191 [3].

All animal studies were conducted and animals handled in accordance with guidelines approved by the Institutional Animal Care and Use Committees at The University of Texas MD Anderson Cancer Center. Female NOD-scid IL2Rγnull-3/GM/SF (NSG-SGM3, NSGS) mice (Jackson Laboratory, Maine, USA, 6-8 weeks old) were injected intravenously with cells isolated from Ph-like B-ALL samples (1×10^6^ cells/100μL/mouse). Four-twelve weeks after injection, engraftment of human CD45/CD19 cells was determined in peripheral blood samples by flow cytometry. Details of the samples are summarized in Table 2. Once tumor burden in peripheral blood was determined to be high (>90% CD45+ CD19+) or the mice presented tumor burden symptoms according to the Institutional Animal Care and Use Committee protocol, mice were humanely sacrificed and their spleens were harvested for *ex vivo* cell killing and cell signaling assays. The collected cells were seeded at 0.4×10^6^ in 0.4ml medium in 48-well plates pre-coated with 200 MS-5 cells and treated with each inhibitor or one of their combinations for 72 h. The cytotoxicity was determined by annexin V/DAPI flow cytometry after gating on CD19+ cells. The specific cell killing was calculated by subtracting spontaneous cell death in DMSO–treated control cells from selected inhibitor-induced cell killing.

### *In vivo* efficacy study

Mice were randomized into treatment groups based on the engraftment level determined by hCD45 flow cytometry. AZD2014 (30mg/kg/d by gavage) was formulated in 20% Captisol (Ligand Techonology, CA, USA). Ruxolitinib was formulated in chow, which was dosed at 2 g/mouse/day. BBT594 (100 mg/kg/d by gavage) was formulated in 0.5% methyl cellulose and 0.2% Tween 80. Upon high PB engraftment (>20%), mice were dosed with inhibitors for 7 days total, on a 3 days on - 2 days off - 4 days on schedule. Animals were humanely sacrificed after last dose, and BM and spleens collected for leukemia burden analysis by hCD45/CD19 flow cytometry.

### Statistical analysis

All statistical analyses were performed using Prism software 7.0. Unless indicated, the results are expressed as the mean± standard deviation of at least three independent experiments. Differences with a *p*-value <0.05 were considered statistically significant.

## SUPPLEMENTARY MATERIALS FIGURES


