# Supplementary material for: Inhibition of mTORC1/C2 signaling improves anti-leukemia efficacy of JAK/STAT blockade in CRLF2 rearranged and/or JAK driven Philadelphia chromosome–like acute B-cell lymphoblastic leukemia
Source: Oncotarget. 2018 Jan 17;9(8):8027–41. doi: 10.18632/oncotarget.24261 (PMC5814279; doi:10.18632/oncotarget.24261)
Supplement: Supplementary file 1 [file oncotarget-09-8027-s001.pdf]

Inhibition of mTORC1/C2 signaling improves anti-leukemia efficacy of JAK/STAT blockade in *CRLF2* rearranged and/or *JAK* driven Philadelphia chromosome–like acute B-cell lymphoblastic leukemia

SUPPLEMENTARY MATERIALS

Cell culture

Murine interleukin 3–dependent pro-B BaF/3 cells expressing Ph-like B-ALL–associated *JAK* mutations were described previously [1]. Specifically, ruxolitinib-persistent cells were generated by culturing BaF/3 cell lines with the different ALL-associated mutations with ruxolitinib for 4 to 6 weeks.[1] REH, Ph-like B-ALL MHH-CALL-4 and MUTZ-5 cells were obtained from DSMZ (Braunschweig, German). All cells were maintained in RPMI-1640 medium with fetal bovine serum (FBS; 20% for MHH-CALL-4 and MUTZ-5, 10% for BaF/3 and REH) and 1% penicillin/streptomycin/10% L-glutamine in a 37°C incubator with 5% CO_2. Human embryonic kidney 293T cells and MS-5 cells were obtained from ATCC (VA, US) and maintained in Dulbecco modified essential medium (DMEM) with FBS (10% for 293T, 20% for MS-5). Cells' identity was validated by tandem repeat DNA finger printing using Amp-FISTR Identifier kit according to the manufacture's instruction. For experiments, the cells were harvested in the logarithmic phase with >85% viability. Different *4E-BP1* expressing cells were constructed as described previously [2]. Briefly, the plasmid constructs and the packaging plasmids were transfected into 293T cells before collecting the virus at 48 h. Then the MHH-CALL4 cells were infected with the virus and cultured under puromycin selection until the stable *4E-BP1* expressing cells were established.

Reagents

Ruxolitinib, rapamycin and AZD2014 for *in vitro* studies were purchased from Selleck Chem (TX, USA). BBT-594 was a gift from T. Radimerski (Novartis, NY, USA). Each inhibitor was dissolved in dimethyl sulfoxide (DMSO) to the final concentration of 10mM. Each stock solution was diluted to the indicated final concentration in the medium. Doxycycline and puromycin were purchased from Sigma (St Louis, MO). Ruxolitinib chow for *in vivo* studies was kindly provided by Incyte (Wilmington, DE). AZD2014 for *in vivo* studies was synthesized by WuXi AppTec (Tianjin, China).

Proliferation and cytotoxicity assays

Cells were incubated with DMSO, ruxolitinib, BBT-594, rapamycin, AZD2014, or a two-drug combination in 96-well plates for 72 h, and viability was determined by the Cell Titer-Glo Luminescent Cell Viability Assay (CTG; Promega, Fitchburg, WI). Experiments were perfromed at least three times, and each experiment was done in quadruplicate. Dose-response curves and plots were generated by non-linear regression with Prism software 6.0 (GraphPad, La Jolla, CA). The combination index (CI) was calculated according to the median-effect principle by the Chou and Talalay method [3], using CalcuSyn Software v2.0 (Premier Biosoft, Palo Alto, CA). A CI less than 0.9 indicates a synergistic effect of the two compounds, a CI 0.9-1.1 is considered additive, a CI greater than 1.1 indicates an antagonistic effect.

Cell apoptosis and cell cycle analyses

Quantification of apoptotic cells after 72 h of drug exposure was accomplished by flow cytometry with annexin V (Roche Diagnostics, Indianapolis, IN) staining for 15 min. 4, 6-diamidino-2-phenylindole dihydrochloride (DAPI) was added immediately before analysis by Gallios flow cytometer (Beckman Coulter, Brea, CA).

For cell cycle distribution analysis, cells (1×10^6) were harvested after drug treatment for 72h, washed twice with phosphate-buffered saline solution (PBS), fixed in cold 95% ethanol containing 10µg/mL RNase for 15 min, and stained with 50µg/mL propidium iodide for 15 min before flow cytometry analysis. Data were processed with FlowJo software v10 (Tree Star, Ashland, OR).

Phospho-flow cytometry

Cells were starved in serum-free Iscove modified Dulbecco medium (IMDM) containing 1% bovine serum albumin (BSA) for 16 h (cell lines) or 1 h (primary samples) before the experiment, then incubated with 0.1% DMSO or one of the following for 1 h: 1µM ruxolitinib, 1 µM BBT-594, 0.2 µM rapamycin, or 0.2 µM AZD2014 or with a combination of a JAK2 inhibitor and an mTOR

inhibitor. Leukemia cells were subsequently stimulated with recombinant human thymic stromal lymphopoietin (TSLP, 25ng/mL, R&D Systems, Minneapolis, MN) for an additional 30 min. After treatment, samples were immediately fixed with paraformaldehyde and subjected to permeabilization with ice-cold 90% methanol (Fisher Scientific, Hampton, NH) overnight. Samples were then washed and stained with surface and phosphoprotein antibodies against human CD45, CD19, and p-STAT5, p-JAK2, p-4E-BP1, p-AKT, p-S6, or p-ERK. Flow cytometry was performed on the Gallios flow cytometer (Beckman Coulter, IN). The data were processed with FlowJo software v10. The heat map was generated by normalized median fluoresce intensity using the Z-Score method with Prism software v7.

Western blotting and revere phase protein array (RPPA) analysis

Cells were starved in serum-free IMDM containing 1% BSA for 16 h and exposed to the agents at the same concentrations and time periods as for the phospho-flow cytometry experiments. Cells were washed once with cold PBS and then subjected to lysis in RIPA cell lysis buffer (Cell Signaling Technology, Beverly, MA) supplemented with 1% protease and phosphatase inhibitors (Cell Signaling Technology). Lysates were separated via sodium dodecyl sulfate–polyacrylamide gel electrophoresis and immunoblotted onto nitrocellulose membranes for probing with the following antibodies overnight at 4°C: STAT5, p-STAT5(Y694), ERK, p-ERKT42/44, AKT, p-AKT(Ser473), S6, p-S6(S240/244), 4E-BP1, p-4E-BP1(T37/46), JAK2, p-JAK2(Tyr1008), eIF4E (all from Cell Signaling Technology), p-eIF4E(S209) (Invitrogen), and β-actin (Sigma). The membranes were then incubated with 680-conjugated donkey anti-rabbit IgG (Invitrogen) and 800-conjugated donkey anti-mouse IgG antibody (Invitrogen) for 1 h. The fluorescence signals were read by the Odyssey imaging system (LI-COR Biosciences, Lincoln, NE). Data were processed by Image Studio software (LI-COR Biosciences).

RPPA was performed at MDACC Core Facility using all antibodies available in the Core. RPPA methodology was described previously [4].

REFERENCES

1. Weigert O, Lane AA, Bird L, Kopp N, Chapuy B, van Bodegom D, Toms AV, Marubayashi S, Christie AL, McKeown M, Paranal RM, Bradner JE, Yoda A, et al. Genetic resistance to JAK2 enzymatic inhibitors is overcome by HSP90 inhibition. J Exp Med. 2012; 209: 259-73. https://doi.org/10.1084/jem.20111694.
2. So L, Lee J, Palafox M, Mallya S, Woxland CG, Arguello M, Truitt ML, Sonenberg N, Ruggero D, Fruman DA. The 4E-BP-eIF4E axis promotes rapamycin-sensitive growth and proliferation in lymphocytes. Sci Signal. 2016; 9: ra57. https://doi.org/10.1126/scisignal.aad8463.
3. Chou TC. Drug combination studies and their synergy quantification using the Chou-Talalay method. Cancer Res. 2010; 70: 440-6. https://doi.org/10.1158/0008-5472.CAN-09-1947.
4. Konopleva MY, Walter RB, Faderl SH, Jabbour EJ, Zeng Z, Borthakur G, Huang X, Kadia TM, Ruvolo PP, Feliu JB, Lu H, Debose L, Burger JA, et al. Preclinical and early clinical evaluation of the oral AKT inhibitor, MK-2206, for the treatment of acute myelogenous leukemia. Clin Cancer Res. 2014; 20: 2226-35. https://doi.org/10.1158/1078-0432.CCR-13-1978.

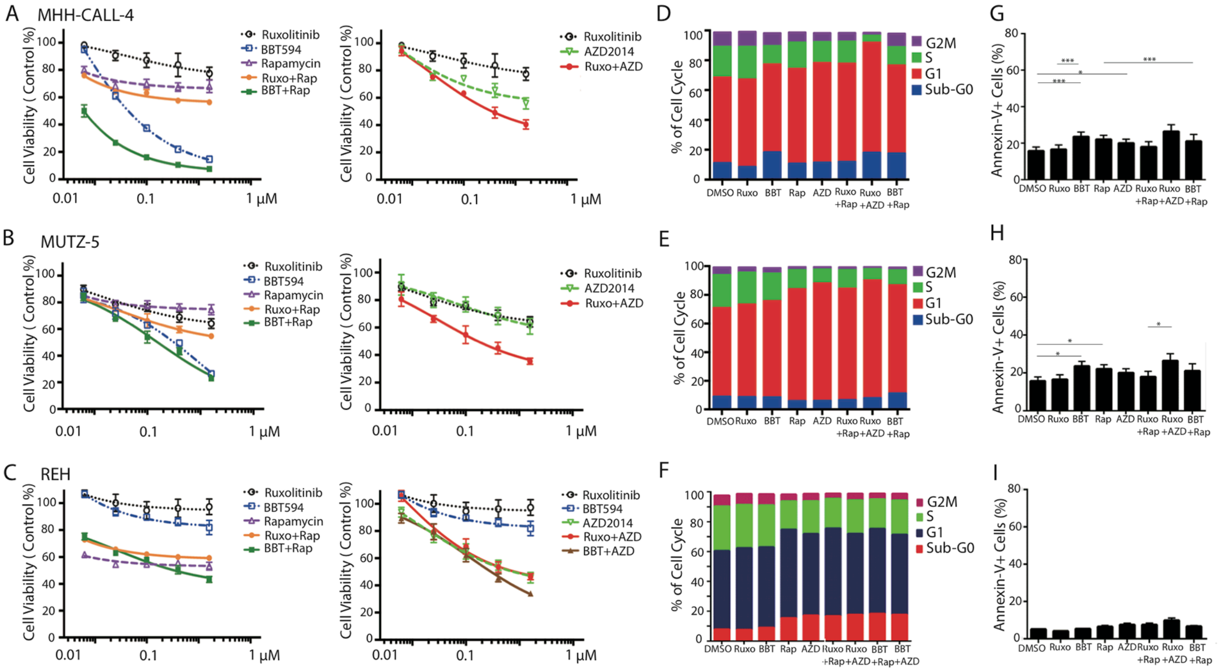

Supplementary Figure 1: ***in vitro*** **antileukemia efficacy of dual JAK2 and mTOR inhibition in Ph-like B-ALL cell lines and in BaF/3 cells with Ph-like B-ALL–associated JAK mutations.** MHH-CALL4 and MUTZ-5 cells were treated with 0.25-0.8µM ruxolitinib (Ruxo), BBT594 (BBT), rapamycin (Rap), AZD2014 (AZD), or one of their combinations for 72 h, then the numbers of viable cells were determined by CTG assay. The cell inhibition curves were plotted with the live cell number normalized to those of DMSO-treated controls: **(A)** MHH-CALL4 cells, **(B)** MUTZ-5 cells, **(C)** REH cells. Treated cells were fixed in 90% methanol and then stained with propidium iodine to determine the effects of the treatments on the cell cycle by flow cytometry: **(D)** MHH-CALL4 cells, **(E)** MUTZ-5 cells, **(F)** REH cells. The cells were stained with annexinV/DAPI to quantify cell apoptosis by flow cytometry: **(G)** MHH-CALL4 cells, **(H)** MUTZ-5 cells, **(I)** REH cells. $^{*}p<0.05$, $^{**}p<0.005$, $^{***}p<0.0005$ as determined by unpaired Student t-test.

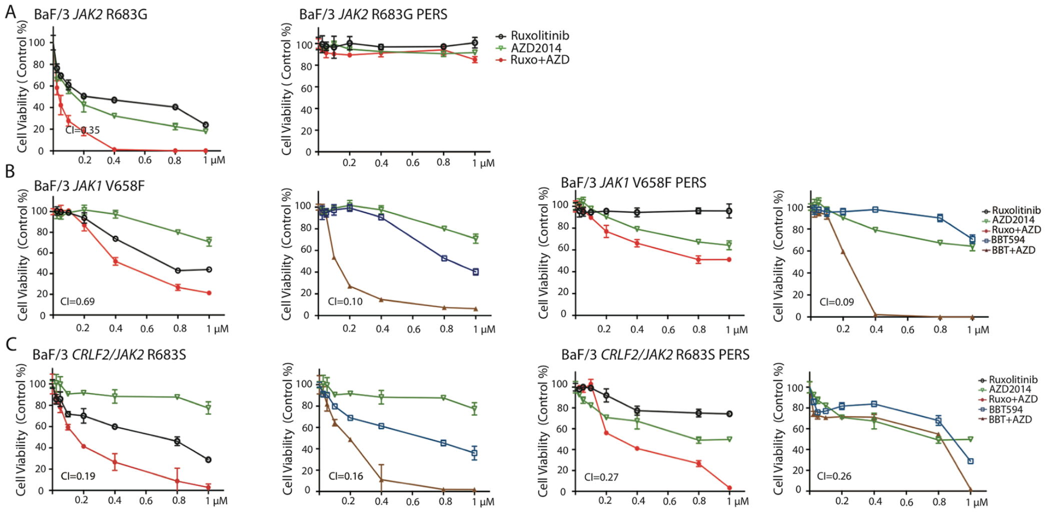

Supplementary Figure 2: *in vitro* antileukemia efficacy of dual JAK2 and mTOR inhibition in BaF/3 cells with Ph-like B-ALL–associated JAK mutations. BaF/3 cells expressing the Ph-like B-ALL-associated *JAK2* R683G **(A)**, *JAK2* V658F **(B)**, or *CRFL2*/*JAK2* R683S **(C)** parental and respective ruxolitinib-"persistent" (PERS) cells (*JAK2* R683G PERS, *JAK2* V658F PERS, and *CRFL2*/*JAK2* R683S PERS) were treated with 0.025-1.0μM ruxolitinib, BBT594, AZD2014 or one of their combinations for 72 h, and cell viability was analyzed by CTG assay. The viability of cells treated was selected inhibitors was to those of DMSO-treated controls, and expressed as % viable cells.
